# Identification of novel prognostic markers of survival time in high-risk neuroblastoma using gene expression profiles

**DOI:** 10.18632/oncotarget.27808

**Published:** 2020-11-17

**Authors:** Abdulazeez Giwa, Azeez Fatai, Junaid Gamieldien, Alan Christoffels, Hocine Bendou

**Affiliations:** ^1^SAMRC Bioinformatics Unit, South African National Bioinformatics Institute, University of the Western Cape, Bellville, South Africa; ^2^Department of Biochemistry, Lagos State University, Lagos, Nigeria

**Keywords:** neuroblastoma, differential gene expression, prognostic markers, machine learning, gene regulatory networks

## Abstract

Neuroblastoma is the most common extracranial solid tumor in childhood. Patients in high-risk group often have poor outcomes with low survival rates despite several treatment options. This study aimed to identify a genetic signature from gene expression profiles that can serve as prognostic indicators of survival time in patients of high-risk neuroblastoma, and that could be potential therapeutic targets. RNA-seq count data was downloaded from UCSC Xena browser and samples grouped into Short Survival (SS) and Long Survival (LS) groups. Differential gene expression (DGE) analysis, enrichment analyses, regulatory network analysis and machine learning (ML) prediction of survival group were performed. Forty differentially expressed genes (DEGs) were identified including genes involved in molecular function activities essential for tumor proliferation. DEGs used as features for prediction of survival groups included *EVX2*, *NHLH2*, *PRSS12*, *POU6F2*, *HOXD10*, *MAPK15*, *RTL1*, *LGR5*, *CYP17A1*, *OR10AB1P*, *MYH14*, *LRRTM3*, *GRIN3A*, *HS3ST5*, *CRYAB* and *NXPH3*. An accuracy score of 82% was obtained by the ML classification models. SMIM28 was revealed to possibly have a role in tumor proliferation and aggressiveness. Our results indicate that these DEGs can serve as prognostic indicators of survival in high-risk neuroblastoma patients and will assist clinicians in making better therapeutic and patient management decisions.

## INTRODUCTION

Neuroblastoma is the most common extracranial solid tumor in childhood accounting for approximately 15% of pediatric cancer death [1–3]. It develops anywhere along the sympathetic nervous system with 60% of the tumors occurring in the abdomen, commonly in the adrenal gland [4, 5].

Outcomes ranging from spontaneous regression to relentless progression despite extensive therapies indicate the heterogeneity of neuroblastoma [6]. The Children’s Oncology Group (COG) classifies neuroblastoma patients into low, intermediate and high-risk groups. Patients classified in low-risk groups have good outcomes contrary to high-risk groups who present poor outcomes despite extensive therapies [4] and with a disproportionate number dying or suffering profound treatment related morbidities [7, 8]. Tumors in high-risk neuroblastoma patients are often metastatic, resulting in survival rates of less than 50% [1].

Genomic studies associated high-risk neuroblastoma with mutations or alterations in a number of genes, such as ALK, ATRX and TERT [9–12]. Furthermore, genome wide association studies have revealed genetic markers, such as CASC15, LMO1, DUSP2 and BARD1 to be neuroblastoma susceptibility genes [13–21]. However, these genes do not provide information about patient survival.

The objective of our study is to identify a genetic signature from gene expression data that can serve as prognostic indicators of survival time in high-risk neuroblastoma patients and that could be therapeutic targets in that patient category.

## RESULTS

Querying the Xena TARGET dataset returned 20 and 12 SS and LS samples, respectively. Based on the gene expression levels in these samples, the edgeR filterByExpr function removed 35,873 low expressed genes and kept 24,610 genes for downstream analysis. The DGE analysis with DESeq2 identified 40 DEGs between the SS and LS groups, of which 21 genes were upregulated and 19 genes were downregulated. Table 1 shows information about the 40 DEGs.

**Table 1:** Upregulated and downregulated DEGs in SS neuroblastoma samples compared to LS samples

Symbol	Name	log2FC	*p*-adj	Status
SMIM28	small integral membrane protein 28	5.4652	0.0146	Up
EVX2	even-skipped homeobox 2	4.9304	0.0014	Up
NHLH2	nescient helix-loop-helix 2	4.2404	0.0046	Up
PRSS12	serine protease 12	3.4788	0.0168	Up
POU6F2	POU class 6 homeobox 2	3.4534	0.0430	Up
HOXD10	homeobox D10	3.3386	0.0357	Up
MAPK15	mitogen-activated protein kinase 15	3.0939	0.0499	Up
RTL1	retrotransposon Gag like 1	2.8233	0.0241	Up
LGR5	leucine rich repeat containing G protein-coupled receptor 5	2.7453	0.0386	Up
DPY19L2P4	DPY19L2 pseudogene 4	2.6439	0.0187	Up
STRA6	signaling receptor and transporter of retinol STRA6	2.5625	0.0437	Up
MEG9	maternally expressed 9	1.9449	0.0146	Up
LINC01410	long intergenic non-protein coding RNA 1410	1.6411	0.0334	Up
CYP17A1	cytochrome P450 family 17 subfamily A member 1	4.1951	0.0005	Down
OR10AB1P	olfactory receptor family 10 subfamily AB member 1 pseudogene	4.0068	0.0146	Down
MYH14	myosin heavy chain 14	3.7783	0.0317	Down
LRRTM3	leucine rich repeat transmembrane neuronal 3	3.6646	0.0168	Down
GRIN3A	glutamate ionotropic receptor NMDA type subunit 3A	−3.1104	0.0445	Down
HS3ST5	heparan sulfate-glucosamine 3-sulfotransferase 5	−2.9968	0.0168	Down
NBAS	NBAS subunit of NRZ tethering complex	−2.8992	0.0146	Down
FNDC9	fibronectin type III domain containing 9	−2.8611	0.0419	Down
H1-4	H1.4 linker histone, cluster member	−2.8427	0.0146	Down
CRYAB	crystallin alpha B	−2.7802	0.0146	Down
NXPH3	neurexophilin 3	−2.5502	0.0348	Down
MYL3	myosin light chain 3	−2.5310	0.0437	Down
CMYA5	cardiomyopathy associated 5	−2.4531	0.0311	Down
AMIGO2	adhesion molecule with Ig like domain 2	−2.2807	0.0499	Down
SIK1B	salt inducible kinase 1B (putative)	−2.2002	0.0446	Down
EDIL3	EGF like repeats and discoidin domains 3	−2.1682	0.0311	Down
UBC	ubiquitin C	−1.1926	0.0499	Down
lnc-FANCC-1		2.6996	0.0437	Up
lnc-KLHL28-1		2.5450	0.0311	Up
lnc-TBCCD1-4		2.1511	0.0051	Up
lnc-CDC27-8		2.1123	0.0146	Up
lnc-SPG21-45		2.0232	0.0166	Up
AC137695.1		2.0062	0.0146	Up
Lnc-NSUN6-1		1.5671	0.0499	Up
Lnc-ZNF814-1		1.3445	0.0146	Up
Lnc-METRNL-8		−2.8955	0.0311	Down
Lnc-METRNL-1		−2.5951	0.0437	Down

Shown are gene symbols, gene names, fold change (log2FC), *p*-adjusted value and gene expression status. Genes without names are non coding RNAs (LNCipedia GeneIDs) except AC137695.1.

**Figure 1:**
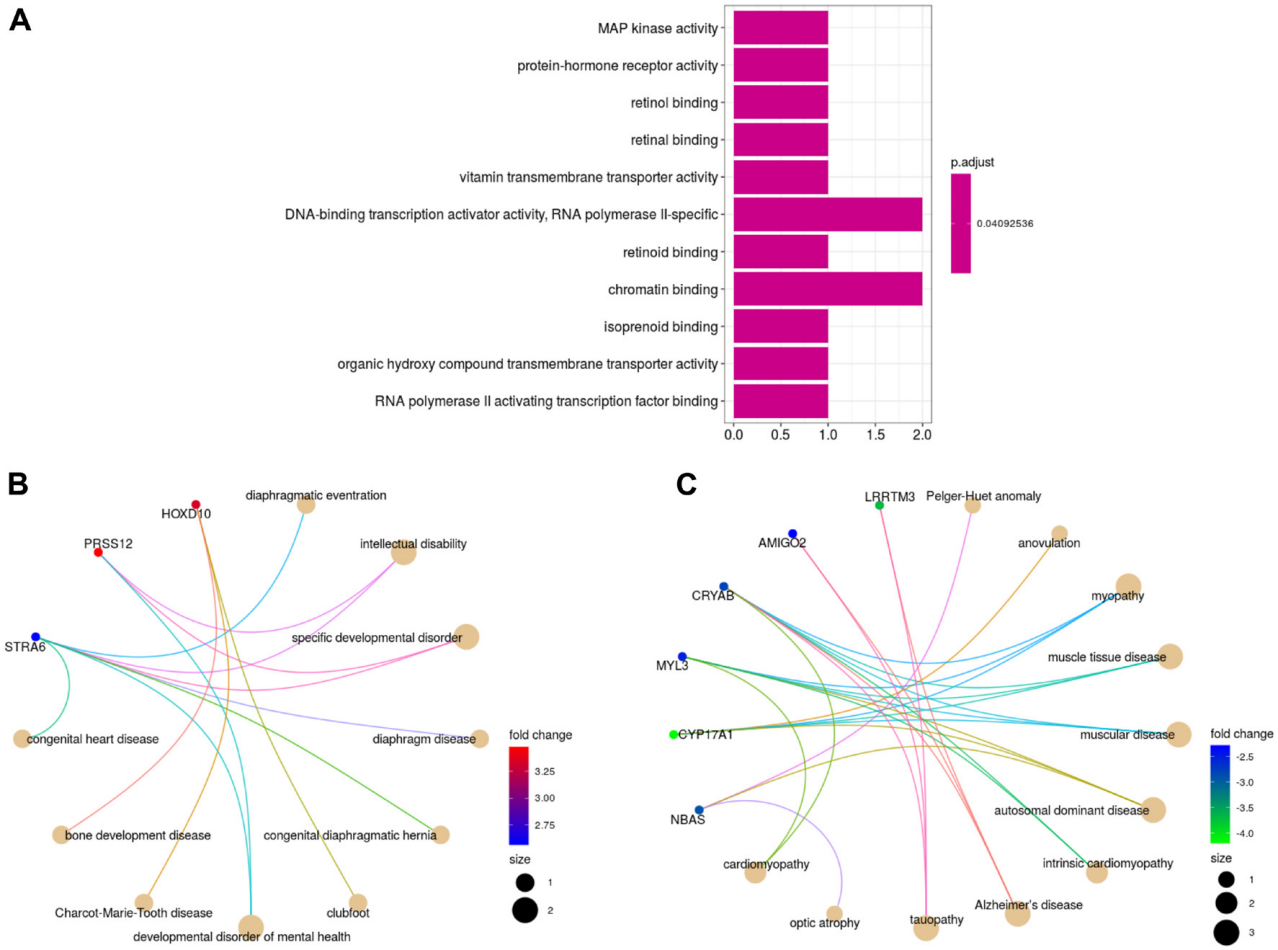
Gene and disease enrichment analyses. Molecular Function activities enriched by the upregulated DEGs (**A**). Network representations of enriched diseases for upregulated DEGs (**B**) and downregulated DEGs (**C**) (absolute value of fold change > 1.5). The size of the circles represents the number of genes that enrich a disease.

The Gene Ontology (GO) Molecular Function enrichment analysis revealed that upregulated genes were mainly enriched in MAP kinase activity, retinol binding and RNA polymerase II activating transcription factor binding, as well as in other activities shown in (Figure 1A). No statistically significant results (*p*-adjusted value < 0.05) were obtained for the downregulated genes as well as for the other GO categories; Biological Process and Cellular Component. In addition, the Disease Ontology enrichment analysis associated upregulated and downregulated genes with several genetic disorders; intellectual disability, cardiac dysfunction, bone development, impaired infertility and pulmonary dysfunction caused by diaphragm-associated abnormalities (Figure 1B and 1C).

**Figure 2:**
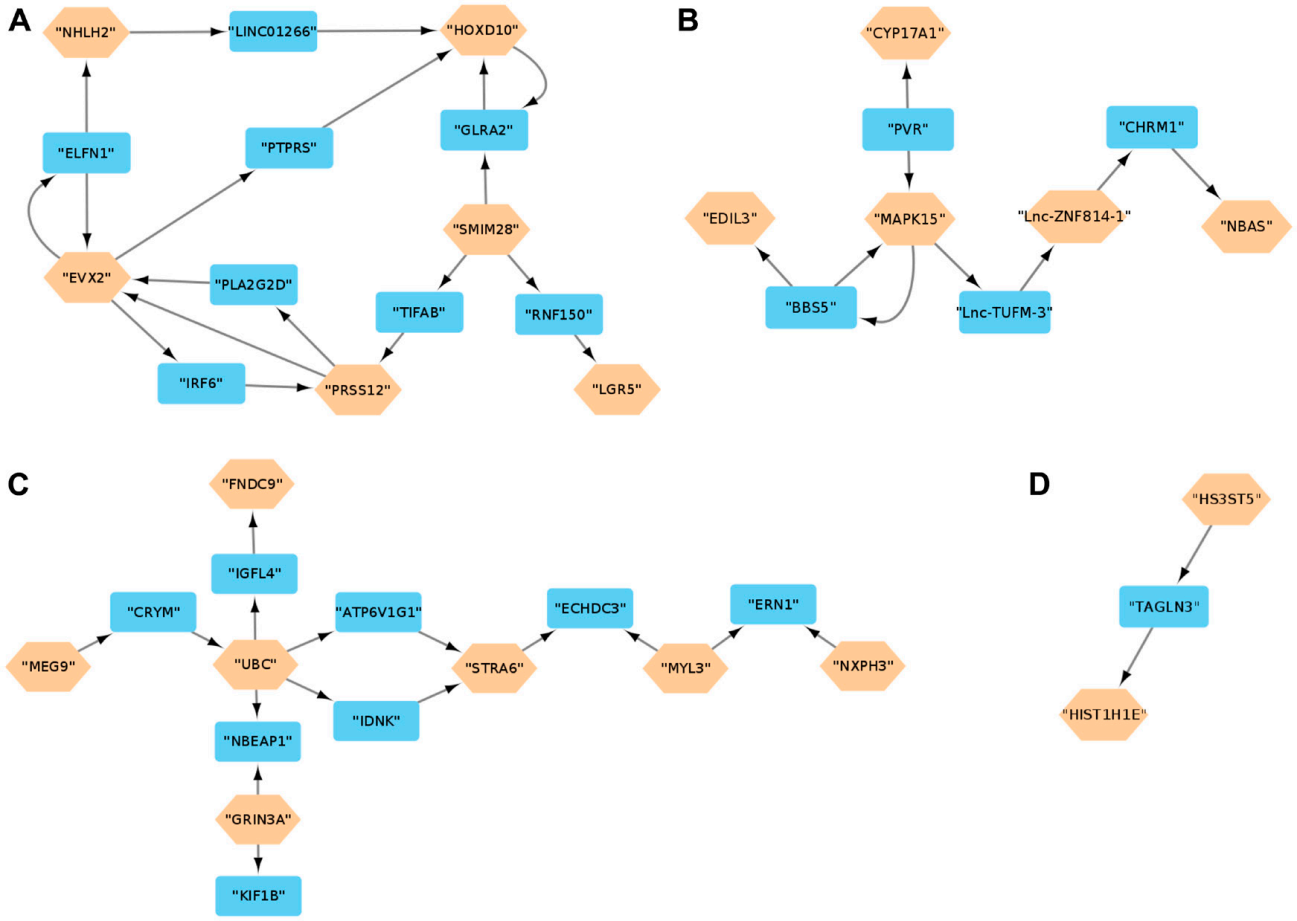
(**A**–**D**) Gene Regulatory Network Analysis. Regulatory networks for the DEG interactions in the SS group. The brown hexagonal nodes are DEGs and the blue rectangles are intermediate genes connecting two DEGs. DEGs that do not interact with other DEGs are omitted.

Reconstruction of gene regulatory networks, using the GENIE3 algorithm, for the SS and LS samples respectively deduced 1,966,606 and 1,967,020 weighted interactions involving the DEGs. Applying a weight threshold value of 0.00251 resulted in 1018 and 650 DEG interactions for the SS and LS groups, respectively. The visualization of the 1018 DEG interactions using Cytoscape enabled the detection of 4 essential regulatory networks (Figure 2). The first network (Figure 2A) involves SMIM28, LGR5, PRSS12, EVX2, NHLH2 and HOXD10. All of these DEGs are upregulated, and the last three genes are transcription factors. The following network (Figure 2B) interconnect MAPK15, Lnc-ZNF814-1, EDIL3, NBAS and CYP17A1. The first two genes are upregulated and the last three genes are downregulated. Most of the DEGs in the third and last networks (Figure 2C and 2D) are downregulated with the exception of MEG9 and STRA6 which are upregulated. Interestingly, these interactions between the DEGs are not present in the LS group and the following genes; SMIM28, HOXD10, PRSS12, NHLH2, MEG9, MAPK15, Lnc-ZNF814-1 and FNDC9, have no interactivity with any other DEG (Supplementary Text 1: *SS-DEG-Net.tsv* and *LS-DEG-Net.tsv* respectively store the 1018 and 650 interactions that can be viewed with Cytoscape).

Filtering the GSE49711 dataset with the query criteria of Table 3 yielded 43 SS and 19 LS samples, respectively. DEGs that do not have an associated *NCBI GeneID* were not found in this dataset, particularly those that are identified as long non-coding RNAs (lncRNAs). Only 25 of the 40 DEGs have expression data in this dataset. Based on the results of feature selection with scikit-learn and several classification tests, the following 16 features were selected for the machine learning construction of the training and test sets; *EVX2, NHLH2, PRSS12, POU6F2, HOXD10, MAPK15, RTL1, LGR5, CYP17A1, OR10AB1P, MYH14, LRRTM3, GRIN3A, HS3ST5, CRYAB and NXPH3*. The evaluation of the Support Vector Machines (SVM) and Artificial Neural Networks (ANN) models using 5-fold cross-validation resulted in an accuracy of 78% and 87% for SVM and ANN, respectively. By testing the ML models on the GSE49711 test set, ANN again achieved better results with an accuracy of 82% of samples correctly classified as SS or LS compared to SVM which obtained an accuracy of 79% (Table 2).

**Table 2:** Machine learning results for the classification of the GSE49711 test set of high-risk neuroblastoma samples with SVM and ANN models

	SVM		ANN	
Class	Precision	Recall	Precision	Recall
Short Survival	0.87	0.81	0.86	0.88
Long Survival	0.64	0.74	0.72	0.68
Accuracy	79%		82%	

**Table 3:** Query criteria for short survival (SS) and long survival (LS) sample selection

Field	SS values	LS values
Diagnostic category	Neuroblastoma	Neuroblastoma
INSS stage	Stage 4	Stage 4
COG risk group	High risk	High risk
Vital status	Dead	Alive
Overall survival time	730 days	2555 days

## DISCUSSION

We aimed at identifying genes that are differentially expressed between high-risk SS and LS patients that could be potential prognostic indicators and or therapeutic targets. The results of the DGE analysis between the two groups showed the upregulation and downregulation of genes associated with neuroblastoma and other cancers.

### Differentially expressed genes

#### Upregulated genes

The upregulated DEGs included some genes whose overexpression in the SS group have been correlated with poor survival in neuroblastoma and other cancers. Higher expression levels of NHLH2 were found to be higher in unfavourable neuroblastomas and was significantly associated with a poor prognosis [22]. Additional roles of NHLH2 in the obesity and fertility has also been uncovered [23, 24]. The upregulation of PRSS12 in this study is similar to the results of Hiyama et al. [25], which reported the overexpression of PRSS12 in neuroblastoma tumors with high telomerase activity correlating with unfavourable tumors. Serine proteases are often altered and significantly upregulated in cancer as malignant cells need proteolytic activities to enable their growth, survival and expansion. Our result support that upregulation of PRSS12 is indicative of poor survival in neuroblastoma. HOXD10 is a transcription factor whose expression is altered in many cancers. Its high expression gives cancer cells proliferative and migratory abilities [26]. Elevated expression of HOXD genes including HOXD10 was reported to be associated with unfavourable prognosis and poor outcome in neuroblastoma [27], which supports our results indicating a more aggressive disease in the SS group. LGR5 is a stem cell marker which is highly expressed and associated with an aggressive phenotype in neuroblastoma [28, 29]. It has also been associated with pancreatic ductal adenocarcinoma [30] and colorectal cancer [31]. LGR5 potentially contributes to stem cell maintenance and self renewal and is indicative of poor survival in high-risk neuroblastoma. SMIM28 is a less studied protein whose upregulation is indicative of poor survival in this study. Similar to our results, Jiang et al. [32] reported the upregulation of SMIM28 in prostate cancer. EVX2 is a homeobox transcription factor essential for vertebrate spinal cord neuronal specification [33]. POU6F2 belongs to the POU class homeobox family whose members are transcriptional regulators and is involved in hereditary predisposition to Wilms tumor, a pediatric malignancy of the kidney [34]. Functional studies are required to elucidate the roles of SMIM28, EVX2 and POU6F2 in high-risk neuroblastoma.

MAPK15, a protein kinase involved in many cellular activity including cell proliferation was upregulated in this study. Highest levels of MAPK15 was found in aggressive embryonal carcinomas and it acts by sustaining the progression of the cell cycle of embryonal carcinomas by limiting p53 activation and preventing the facilitation of p53 dependent mechanisms that results to the arrest of the cell cycle [35]. Neuroblastoma is a malignancy of embryonal origin and upregulation of MAPK15 would be expected to facilitate tumor progression and indicative of aggressive disease and poor survival as in the SS group. RTL1, a paternally expressed imprinted gene highly expressed in the fetus, placenta and brain. It has been reported to be a driver of hepatocellular carcinoma [36]. Being a protease, it possibly promotes tumor invasion and metastasis in neuroblastoma tumors. Higher expression of RTL1 is thus suggestive of poor prognosis in neuroblastoma. Also upregulated is STRA6, a plasma membrane protein that transports retinol and is involved in a signalling mediated by JAK2, STAT3 and STAT5 [37]. Its upregulation indicates poor survival in our study possibly through its maintenance of cancer stem cells and promotion of tumor formation as reported in colorectal cancer [37, 38].

Ten lncRNAs were upregulated in this study. Three of these (lnc-SPG21-45, lnc-NSUN6-1 and lnc-KLHL28-1) are antisense to ANKDD1A, CACNB2 and C14orf28 genes, respectively, which are associated with other cancers and diseases, possibly by regulating their expression. C14orf28 has been observed to be overexpressed in colorectal cancer cells, promoting proliferation, migration and invasion [39]. ANKDD1A has been described as a functional tumor suppressor with germline variants predicting poor patient outcomes in low grade glioma [40], and is frequently methylated in glioblastoma multiforme [41] and in clinically non-functioning pituitary adenomas [42]. CACNB2 is a calcium channel protein linked to diabetic retinopathy [43], bipolar disorder [44], hypertension [45] and autism spectrum disorders [46]. The role of calcium signalling in cancer has been reviewed by [47–49]. MEG9, is located in an imprinted non-coding RNA genomic region, DLK1-DIO3 [50, 51]. LINC01410 is a lncRNA highly expressed in pancreatic cancer tissues and cell lines [52]. High expression in cholangiocarcinoma and gastric cancer patients have been associated with poor prognosis and survival [53, 54]. These lncRNAs may be facilitating the promotion of tumor progression, proliferation and invasion which might have impacted the survival of patients in the short survival group. It is well established that metastasis is the primary cause of cancer mortality. Functional studies are required to evaluate the roles of these lncRNAs in neuroblastoma.

#### Downregulated genes

The downregulated genes include genes such as AMIGO2, LRRTM3, GRIN3A, MYH14, EDIL3, FNDC9, involved in neural function and development, and in extracellular matrix (ECM) organization. AMIGO2 is a transmembrane molecule expressed in neuronal tissues and participates in their formation [55]. EDIL3 is an inducer of the epithelial-mesenchymal transition, that promotes angiogenesis and invasion in hepatocellular carcinoma [56]. FNDC9 which exhibits biased expressed in the brain is an ECM protein involved in tumorigenesis in different cancers [57]. MYH14 is a myosin, an actin-dependent motor protein that plays a role in neuritogenesis. Members of the myosin superfamily have been known to enhance or suppress tumor progression [58]. MYH14 could be suppressing tumor progression in high-risk neuroblastoma. The downregulation of these ECM associated genes could be promoting the invasion and metastasis of neuroblastoma tumors.

OR10AB1P belongs to the olfactory receptor family of genes. Olfactory receptors are expressed in various human tissues and are involved in different cellular processes such as migration and proliferation. Some are biomarkers for prostate, lung and small intestine carcinoma tissues [59]. Decreased expression of CRYAB indicated its tumor suppressor function in bladder cancer [60]. It may thus also be functioning as a tumor suppressor in neuroblastoma. Ubiquitin C (UBC) is a polyubiquitin precursor. Ubiquitination has been associated with many cellular processes which play roles in tumorigenesis.

CYP17A1 is a key enzyme in the steroidogenic pathway with restricted expression in the adrenal gland. Neuroblastoma tumors commonly occur in the adrenal medulla. The downregulation of CYP17A1 may be an indicator of poor prognosis in neuroblastoma. LRRTM3 has high expression in the brain and belongs to a group of proteins involved in nervous system development. How it contributes to neuroblastoma remains to be investigated but it is currently a candidate gene for alzheimer’s disease [61, 62], a neurological disorder. NBAS is thought to be involved in golgi-to-ER transport and is typically amplified in MYCN-amplified neuroblastoma tumors [63]. GRIN3A is a glutamate receptor that promotes nerve outgrowth [64]. The downregulation of GRIN3A may suggest a higher level of disease because glutamate is a major excitatory neurotransmitter in the CNS that is involved in many neuronal processes. Functional studies are required to investigate how these genes contribute to poor survival in neuroblastoma.

### Gene and disease ontology enrichment analyses

The molecular function activities; MAP kinase activity, retinol binding and RNA polymerase II activating transcription factor binding, enriched by the upregulated genes MAPK15, STRA16 and NHLH2, respectively, are activities that promote tumor cell proliferation. Deregulation of the MAPK signalling was associated with cancer development, progression and cell proliferation [65, 66]. Retinol binding through the STRA6 upregulation activates a signalling cascade that is found to play a role in cancer initiation, maintenance and growth [38]. Furthermore, the increased global transcription activity (activation of RNA polymerase II) indicates an intensity of a rapid proliferation of cancer cells [67]. These activities again demonstrate the aggressiveness of the neuroblastoma tumors in SS patients compared to LS patients.

The enriched diseases by the upregulated and downregulated genes, particularly the disorders inducing heart failure (Cardiomyopathy and Congenital heart disease) and respiratory illness (Diaphragmatic dysfunction) may have negative impact on survival [68, 69], and could be the cause of death in the SS neuroblastoma patients. There is no indication in the clinical information accompanying the gene expression count dataset if the patients suffered from additional disorders in addition to neuroblastoma. However, it is reported that high-risk neuroblastoma survivors treated with intensive multimodality therapy are at risk for a broad variety of treatment-related late effects including cardiac dysfunction, bone development disease, pulmonary dysfunction and impaired fertility [70]. These treatment-related morbidities were enriched by the upregulated and downregulated DEGs (Figure 1B and 1C), suggesting that the cause of death in patients with SS neuroblastoma may be due to treatment complications.

### Regulatory network analysis

After the application of a weight threshold of 0.00251 to the GENIE3 output, the numbers of interactions involving the DEGs in the SS and LS groups were 1018 and 650, respectively. There is a clear substantial difference in number of interactions between the two groups, indicating a higher cellular/tumor activity in the SS group which could be a sign of tumor aggressiveness in patients with SS neuroblastoma. In addition, it is noticeable from the networks in Figure 2 the importance of the genes SMIM28, MAPK15 and UBC as origins of most of the interactions. The role of MAPK15 and UBC in tumorigenesis has been reported in many previously discussed scientific works, while SMIM28 is a less studied gene with an unclear role in cancer. However, observation of the role of the final target genes of SMIM28 in Figure 2A network, which are LGR5 and HOXD10, can shed light on the role of this gene. As reported previously, both LGR5 and HOXD10 were associated with cancer cell proliferation and tumor aggressiveness in neuroblastoma. Thus, it is possible that SMIM28 (Small Integral Membrane Protein 28) is part of a signalling pathway whose role is to accelerate neuroblastoma tumor proliferation, making it a possible new gene target therapy in high-risk neuroblastoma cancer. Further investigations are required to elucidate precisely the role of SMIM28 in the aggressiveness of neuroblastoma tumors.

### Machine learning

Although not all the DEGs were included in the training and testing of the machine learning models, the obtained prediction results were significantly good. The ANN model obtained the highest accuracy of 82% for the classification of the external neuroblastoma samples (GSE49711) into short and long survival classes. This high classification accuracy makes it possible to consider that the DEG expression profiles have been preserved in the various high-risk neuroblastoma tumors, although neuroblastoma is known to be heterogeneous. Therefore, the DEG list can serve as prognostic indicators (genetic signature) for survival time in high-risk neuroblastoma patients and can be targets for drug discovery analyses. Relatively similar to our study, other studies have proposed genetic signatures for prognostic stratification of patients with neuroblastoma. Liu et al. [71] used an unsupervised biclustering machine learning technique to find high-risk neuroblastoma subtypes. They proposed a signature of 238 neuroblastoma-specific immune genes to identify ultra high-risk and high-risk neuroblastoma subtypes. Russo et al. [72] applied K-means clustering to 27 kinome gene signature to identify ultra high-risk subtypes of high-risk neuroblastoma. Formicola et al. [73] used Cox regression and Kaplan-Meier analysis methods to propose a 18-gene expression based risk scoring system to predict overall survival of patients with stage 4 neuroblastoma. We demonstrated the use of a shorter signature in a 16-gene expression classifier based on survival time to stratify high-risk neuroblastomas into SS (ultra high-risk) and LS subtypes. The number of genes in our classifier should provide the advantage of being less costly and easier to implement.

## MATERIALS AND METHODS

**Figure 3:**
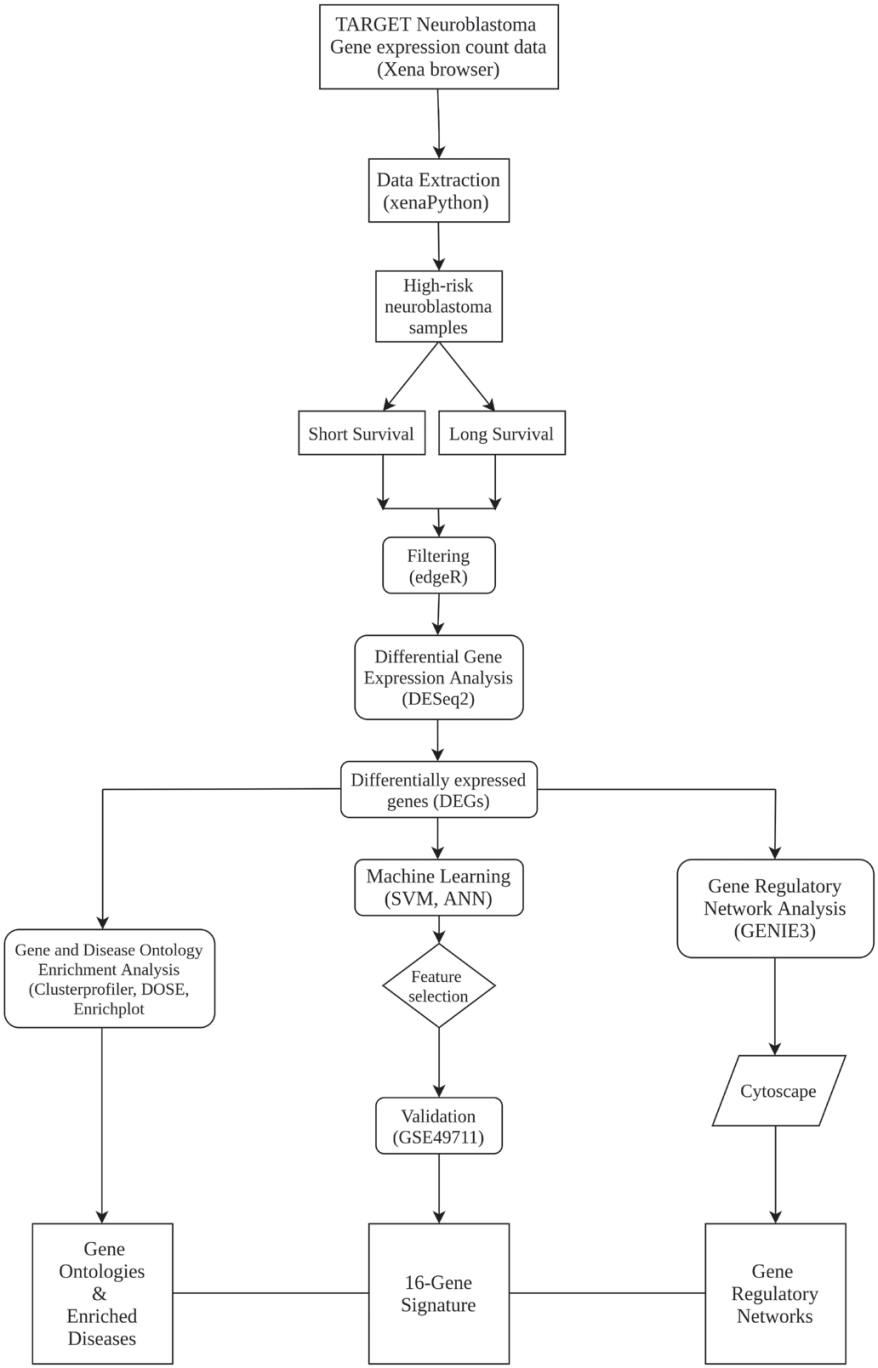
Workflow outlining the steps and methods undertaken in this study.

The workflow describing the steps and methods undertaken in this study is illustrated in Figure 3. It includes 5 essential steps; dataset retrieval, differential gene expression, disease/gene ontology enrichment, gene regulatory network inference and machine learning.

### Datasets

The Therapeutically Applicable Research to Generate Effective Treatments (TARGET) initiative employed comprehensive molecular characterization of hard-to-treat childhood cancers which included neuroblastoma. TARGET data is accessible via the TARGET data matrix as well as via the Xena browser. The Xena browser is a web based visualization and exploration tool for multi-omic data large public repositories and private datasets [74]. The TARGET neuroblastoma dataset in the Xena database [74, 75] is composed of high-risk neuroblastoma samples with available clinical information. Gene expression RNA-seq read counts of the TARGET neuroblastoma dataset (dataset ID: TARGET-NBL. htseq_counts. tsv) were obtained from the GDC hub in Xena browser using xenaPython package. Fields used in querying the dataset are described in Table 3. The dataset itself was composed of 151 samples in total. Querying the dataset with the above fields returned 32 neuroblastoma samples; 20 of which with an overall survival time of less than 730 days (2 years) and vital status is “dead” were considered short survival (SS), while 12 samples with an overall survival time greater than 2555 days (7 years) and vital status is “alive” were considered long survival (LS).

### Differential gene expression analysis

Normalized expression counts were converted to raw counts and filtered to remove low expressed genes using the edgeR filterByExpr function in R [76]. We then performed a differential gene expression (DGE) analysis between the short and long survival groups using the DESeq2 package in R [77]. DESeq2 uses shrinkage estimators for dispersion and fold change for its comparative differential gene expression estimation [77]. Differentially expressed genes (DEGs) were selected by meeting criteria of adjusted *p-value <* 0.05. Gene Ontology (GO) enrichment analysis was carried out to functionally annotate the DEGs using clusterProfiler [78] and visualized using enrichplot. The DOSE R library [79] was used to detect the diseases enriched by the upregulated and downregulated DEGs.

### Machine learning

Scikit-learn [80] and LibSVM [81] libraries were used for machine learning (ML) model creation and classification tasks. Features (genes) not present in the test dataset (GSE49711) were not used for machine learning prediction. The top upregulated and downregulated genes used as features for machine learning (ML) prediction of patient survival were; *EVX2, NHLH2, PRSS12, POU6F2, HOXD10, MAPK15, RTL1, LGR5, CYP17A1, OR10AB1P, MYH14, LRRTM3, GRIN3A, HS3ST5, CRYAB, NXPH3*. The features were extracted from the log-normalized counts data. For LibSVM, the feature values of the training and test sets were scaled using a built-in python script. With regards to ANN, the feature values of the training and test sets were scaled with the Scikit-learn MinMaxScaler function. Algorithms used for training and evaluation of the models include; Support Vector Machines (SVM) and Artificial Neural Networks (ANN). 5-fold cross validation was done to determine the best parameters of the SVM and ANN models which was then applied to our test samples. Both models, created with LibSVM and ANN were applied to predict the classification of samples in an external dataset (GSE49711) with same sample characteristics (overall survival < 730 days with vital status as dead for SS samples, and overall survival > 2555 days with vital status as alive for LS samples). The evaluation metrics for the LibSVM and ANN models were precision, recall and accuracy.

### Regulatory networks

The GENIE3 package [82] in R was used for genetic regulatory network inference analysis. The GENIE3 algorithm uses a Random Forest or Extra Randomized Trees approach to infer gene regulatory networks from gene expression data [82]. It outputs a ranked list of each pairwise comparison from the most to the least confident regulatory connection. The library was run on the gene expression counts from the SS and LS samples separately. For the output analysis, only connections involving the DEGs were considered. In addition, a weighting threshold of 0.00251 was applied to reduce the large number of connections and also to focus on high confident regulatory connections. Cytoscape [83] was used for the visualization of gene regulatory networks.

## CONCLUSIONS

The DGE analysis is a powerful technique for identifying DEGs in a studied condition. In this study, using DESeq2 we identified 40 DEGs between SS and LS neuroblastoma samples. Many of the DEGs were found to be related to different cancers (including neuroblastoma), thus strengthening their possibility of being associated with neuroblastoma. The ML models based on 16 DEGs were capable of stratifying high-risk neuroblastoma samples on the basis of survival time, demonstrating their ability to be used as a genetic signature or prognostic indicators of survival in high-risk neuroblastoma patients. This study furthers our understanding of the molecular mechanisms of neuroblastoma in high-risk patients. We identified SMIM28 gene to be critical for tumor proliferation making it as a possible gene therapy target. Nevertheless, additional studies are required to elucidate the role of SMIM28 in the pathogenesis of neuroblastoma. Finally, prognostic stratification of high-risk neuroblastoma patients will help clinicians in making better therapeutic and patient management decisions.

## SUPPLEMENTARY MATERIALS





## Abbreviations

DGE: Differential Gene Expression
DEG: Differentially Expressed Gene
GO: Gene Ontology
DO: Disease Ontology
ML: Machine Learning
ANN: Artificial Neural Networks
SVM: Support Vector Machines
TARGET: Therapeutically Applicable Research to Generate Effective Treatments

## References

[1] Maris JM . Recent Advances in Neuroblastoma. N Engl J Med. 2010; 362:2202–2211. 10.1056/NEJMra0804577. 20558371 PMC3306838

[2] Smith MA , Seibel NL , Altekruse SF , Ries LA , Melbert DL , O’Leary M , Smith FO , Reaman GH . Outcomes for children and adolescents with cancer: challenges for the twenty-first century. J Clin Oncol. 2010; 28:2625–2634. 10.1200/JCO.2009.27.0421. 20404250 PMC2881732

[3] Ward E , DeSantis C , Robbins A , Kohler B , Jemal A . Childhood and adolescent cancer statistics, 2014. CA Cancer J Clin. 2014; 64:83–103. 10.3322/caac.21219. 24488779

[4] Zhang L , Lv C , Jin Y , Cheng G , Fu Y , Yuan D , Tao Y , Guo Y , Ni X , Shi T . Deep learning-based multi-omics data integration reveals two prognostic subtypes in high-risk neuroblastoma. Front Genet. 2018; 9:477. 10.3389/fgene.2018.00477. 30405689 PMC6201709

[5] Johnsen JI , Dyberg C , Wickström M . Neuroblastoma—A Neural Crest Derived Embryonal Malignancy. Front Mol Neurosci. 2019; 12:9. 10.3389/fnmol.2019.00009. 30760980 PMC6361784

[6] Baali I , Acar DAE , Aderinwale TW , HafezQorani S , Kazan H . Predicting clinical outcomes in neuroblastoma with genomic data integration. Biol Direct. 2018; 13:20. 10.1186/s13062-018-0223-8. 30621745 PMC6889397

[7] Oeffinger KC , Mertens AC , Sklar CA , Kawashima T , Hudson MM , Meadows AT , Friedman DL , Marina N , Hobbie W , Kadan-Lottick NS , Schwartz CL , Leisenring W , Robison LL , et al. Chronic health conditions in adult survivors of childhood cancer. N Engl J Med. 2006; 355:1572–1582. 10.1056/NEJMsa060185. 17035650

[8] Yu AL , Gilman AL , Ozkaynak MF , London WB , Kreissman SG , Chen HX , Smith M , Anderson B , Villablanca JG , Matthay KK , Shimada H , Grupp SA , Seeger R , et al. Anti-GD2 antibody with GM-CSF, interleukin-2, and isotretinoin for neuroblastoma. N Engl J Med. 2010; 363:1324–1334. 10.1056/NEJMoa0911123. 20879881 PMC3086629

[9] Mossé YP , Laudenslager M , Longo L , Cole KA , Wood A , Attiyeh EF , Laquaglia MJ , Sennett R , Lynch JE , Perri P , Laureys G , Speleman F , Kim C , et al. Identification of ALK as a major familial neuroblastoma predisposition gene. Nature. 2008; 455:930–935. 10.1038/nature07261. 18724359 PMC2672043

[10] Valentijn LJ , Koster J , Zwijnenburg DA , Hasselt NE , van Sluis P , Volckmann R , van Noesel MM , George RE , Tytgat GA , Molenaar JJ , Versteeg R . TERT rearrangements are frequent in neuroblastoma and identify aggressive tumors. Nat Genet. 2015; 47:1411–1414. 10.1038/ng.3438. 26523776

[11] Cheung NK , Zhang J , Lu C , Parker M , Bahrami A , Tickoo SK , Heguy A , Pappo AS , Federico S , Dalton J , Cheung IY , Ding L , Fulton B , et al. Association of Age at Diagnosis and Genetic Mutations in Patients with Neuroblastoma. JAMA. 2012; 307:1062–1071. 10.1001/jama.2012.228. 22416102 PMC3527076

[12] Peifer M , Hertwig F , Roels F , Dreidax D , Gartlgruber M , Menon R , Krämer A , Roncaioli JL , Sand F , Heuckmann JM , Ikram F , Schmidt R , Ackermann S , et al. Telomerase activation by genomic rearrangements in high-risk neuroblastoma. Nature. 2015; 526:700–704. 10.1038/nature14980. 26466568 PMC4881306

[13] Maris JM , Mosse YP , Bradfield JP , Hou C , Monni S , Scott RH , Asgharzadeh S , Attiyeh EF , Diskin SJ , Laudenslager M , Winter C , Cole KA , Glessner JT , et al. Chromosome 6p22 locus associated with clinically aggressive neuroblastoma. N Engl J Med. 2008; 358:2585–2593. 10.1056/NEJMoa0708698. 18463370 PMC2742373

[14] Cimmino F , Avitabile M , Diskin SJ , Vaksman Z , Pignataro P , Formicola D , Cardinale A , Testori A , Koster J , de Torres C , Devoto M , Maris JM , Iolascon A , et al. Fine mapping of 2q35 high-risk neuroblastoma locus reveals independent functional risk variants and suggests full-length BARD1 as tumor-suppressor. Int J Cancer. 2018; 143:2828–2837. 10.1002/ijc.31822. 30132831 PMC6258207

[15] Capasso M , Diskin S , Cimmino F , Acierno G , Totaro F , Petrosino G , Pezone L , Diamond M , McDaniel L , Hakonarson H , Iolascon A , Devoto M , Maris JM . Common genetic variants in NEFL influence gene expression and neuroblastoma risk. Cancer Res. 2014; 74:6913–6924. 10.1158/0008-5472.CAN-14-0431. 25312269 PMC4253722

[16] Diskin SJ , Hou C , Glessner JT , Attiyeh EF , Laudenslager M , Bosse K , Cole K , Mosse YP , Wood A , Lynch JE , Pecor K , Diamond M , Winter C , et al. Copy number variation at 1q21.1 associated with neuroblastoma. Nature. 2009; 459:987–991. 10.1038/nature08035. 19536264 PMC2755253

[17] Wang K , Diskin SJ , Zhang H , Attiyeh EF , Winter C , Hou C , Schnepp RW , Diamond M , Bosse K , Mayes PA , Glessner J , Kim C , Frackelton E , et al. Integrative genomics identifies LMO1 as a neuroblastoma oncogene. Nature. 2011; 469:216–220. 10.1038/nature09609. 21124317 PMC3320515

[18] Bosse KR , Diskin SJ , Cole KA , Wood AC , Schnepp RW , Norris G , Nguyen LB , Jagannathan J , Laquaglia M , Winter C , Diamond M , Hou C , Attiyeh EF , et al. Common variation at BARD1 results in the expression of an oncogenic isoform that influences neuroblastoma susceptibility and oncogenicity. Cancer Res. 2012; 72:2068–2078. 10.1158/0008-5472.CAN-11-3703. 22350409 PMC3328617

[19] Oldridge DA , Wood AC , Weichert-Leahey N , Crimmins I , Sussman R , Winter C , McDaniel LD , Diamond M , Hart LS , Zhu S , Durbin AD , Abraham BJ , Anders L , et al. Genetic predisposition to neuroblastoma mediated by a LMO1 super-enhancer polymorphism. Nature. 2015; 528:418–421. 10.1038/nature15540. 26560027 PMC4775078

[20] Russell MR , Penikis A , Oldridge DA , Alvarez-Dominguez JR , McDaniel L , Diamond M , Padovan O , Raman P , Li Y , Wei JS , Zhang S , Gnanchandran J , Seeger R , et al. CASC15-S is a tumor suppressor lncRNA at the 6p22 neuroblastoma susceptibility locus. Cancer Res. 2015; 75:3155–3166. 10.1158/0008-5472.CAN-14-3613. 26100672 PMC4526355

[21] Tonini GP , Capasso M . Genetic predisposition and chromosome instability in neuroblastoma. Cancer Metastasis Rev. 2020; 39:275–285. 10.1007/s10555-020-09843-4. 31927719

[22] Aoyama M , Ozaki T , Inuzuka H , Tomotsune D , Hirato J , Okamoto Y , Tokita H , Ohira M , Nakagawara A . LMO3 interacts with neuronal transcription factor, HEN2, and acts as an oncogene in neuroblastoma. Cancer Res. 2005; 65:4587–4597. 10.1158/0008-5472.CAN-04-4630. 15930276

[23] Good DJ , Braun T . NHLH2: At the intersection of obesity and fertility. Trends Endocrinol Metab. 2013; 24:385–390. 10.1016/j.tem.2013.04.003. 23684566 PMC3732504

[24] Vella KR , Burnside AS , Brennan KM , Good DJ . Expression of the hypothalamic transcription factor nhlh2 is dependent on energy availability. J Neuroendocrinol. 2007; 19:499–510. 10.1111/j.1365-2826.2007.01556.x. 17532796 PMC3111914

[25] Hiyama E , Hiyama K , Nishiyama M , Reynolds CP , Shay JW , Yokoyama T . Differential gene expression profiles between neuroblastomas with high telomerase activity and low telomerase activity. J Pediatr Surg. 2003; 38:1730–1734. 10.1016/j.jpedsurg.2003.08.042. 14666454

[26] Hakami F , Darda L , Stafford P , Woll P , Lambert DW , Hunter KD . The roles of HOXD10 in the development and progression of head and neck squamous cell carcinoma (HNSCC). Br J Cancer. 2014; 111:807–816. 10.1038/bjc.2014.372. 25010866 PMC4134504

[27] Kocak H , Ackermann S , Hero B , Kahlert Y , Oberthuer A , Juraeva D , Roels F , Theissen J , Westermann F , Deubzer H , Ehemann V , Brors B , Odenthal M , et al. Hox-C9 activates the intrinsic pathway of apoptosis and is associated with spontaneous regression in neuroblastoma. Cell Death Dis. 2013; 4:e586. 10.1038/cddis.2013.84. 23579273 PMC3668636

[28] Vieira GC , Chockalingam S , Melegh Z , Greenhough A , Malik S , Szemes M , Park JH , Kaidi A , Zhou L , Catchpoole D , Morgan R , Bates DO , Gabb PJ , et al. LGR5 regulates pro-survival MEK/ERK and proliferative Wnt/β-catenin signalling in neuroblastoma. Oncotarget. 2015; 6:40053–40067. 10.18632/oncotarget.5548. 26517508 PMC4741879

[29] Forgham H , Johnson D , Carter N , Veuger S , Carr-Wilkinson J . Stem Cell Markers in Neuroblastoma—An Emerging Role for LGR5. Front Cell Dev Biol. 2015; 3:77. 10.3389/fcell.2015.00077. 26697427 PMC4667032

[30] Kuraishi Y , Uehara T , Kobayashi Y , Nakajima T , Watanabe T , Shimizu A , Ota H , Tanaka E . Correlation of clinicopathological features and leucine-rich repeat-containing G-protein-coupled receptor 5 expression in pancreatic ductal adenocarcinoma. Pathol Res Pract. 2019; 215:152623. 10.1016/j.prp.2019.152623. 31543221

[31] Morgan R , Mortensson E , Williams A . Targeting LGR5 in colorectal cancer: therapeutic gold or too plastic? Br J Cancer. 2018; 118:1410–1418. 10.1038/s41416-018-0118-6. 29844449 PMC5988707

[32] Jiang T , Guo J , Hu Z , Zhao M , Gu Z , Miao S . Identification of potential prostate cancer-related pseudogenes based on competitive endogenous RNA network hypothesis. Med Sci Monit. 2018; 24:4213–4239. 10.12659/MSM.910886. 29923546 PMC6042310

[33] Juárez-Morales JL , Schulte CJ , Pezoa SA , Vallejo GK , Hilinski WC , England SJ , de Jager S , Lewis KE . Evx1 and Evx2 specify excitatory neurotransmitter fates and suppress inhibitory fates through a Pax2-independent mechanism. Neural Dev. 2016; 11:5. 10.1186/s13064-016-0059-9. 26896392 PMC4759709

[34] Perotti D , De Vecchi G , Testi MA , Lualdi E , Modena P , Mondini P , Ravagnani F , Collini P , Di Renzo F , Spreafico F , Terenziani M , Sozzi G , Fossati-Bellani F , et al. Germline mutations of the POU6F2 gene in Wilms tumors with loss of heterozygosity on chromosome 7p14. Hum Mutat. 2004; 24:400–407. 10.1002/humu.20096. 15459955

[35] Rossi M , Colecchia D , Ilardi G , Acunzo M , Nigita G , Sasdelli F , Celetti A , Strambi A , Staibano S , Croce CM , Chiariello M . MAPK15 upregulation promotes cell proliferation and prevents DNA damage in male germ cell tumors. Oncotarget. 2016; 7:20981–20998. 10.18632/oncotarget.8044. 26988910 PMC4991506

[36] Riordan JD , Keng VW , Tschida BR , Scheetz TE , Bell JB , Podetz-Pedersen KM , Moser CD , Copeland NG , Jenkins NA , Roberts LR , Largaespada DA , Dupuy AJ . Identification of rtl1, a retrotransposon-derived imprinted gene, as a novel driver of hepatocarcinogenesis. PLoS Genet. 2013; 9:e1003441. 10.1371/journal.pgen.1003441. 23593033 PMC3616914

[37] Berry DC , Levi L , Noy N . Holo-retinol-binding protein and its receptor STRA6 drive oncogenic transformation. Cancer Res. 2014; 74:6341–6351. 10.1158/0008-5472.CAN-14-1052. 25237067 PMC4216741

[38] Karunanithi S , Levi L , DeVecchio J , Karagkounis G , Reizes O , Lathia JD , Kalady MF , Noy N . RBP4-STRA6 pathway drives cancer stem cell maintenance and mediates high-fat diet-induced colon carcinogenesis. Stem Cell Reports. 2017; 9:438–450. 10.1016/j.stemcr.2017.06.002. 28689994 PMC5549806

[39] Yang X , Hu Y , Liu Y , Liu W , Zhao X , Liu M , Tang H . C14orf28 downregulated by miR-519d contributes to oncogenicity and regulates apoptosis and EMT in colorectal cancer. Mol Cell Biochem. 2017; 434:197–208. 10.1007/s11010-017-3049-2. 28455792

[40] Chatrath A , Kiran M , Kumar P , Ratan A , Dutta A . The germline variants rs61757955 and rs34988193 are predictive of survival in lower grade glioma patients. Mol Cancer Res. 2019; 17:1075–1086. 10.1158/1541-7786.MCR-18-0996. 30651372 PMC6497557

[41] Feng J , Zhang Y , She X , Sun Y , Fan L , Ren X , Fu H , Liu C , Li P , Zhao C , Liu Q , Liu Q , Li G , et al. Hypermethylated gene ANKDD1A is a candidate tumor suppressor that interacts with FIH1 and decreases HIF1α stability to inhibit cell autophagy in the glioblastoma multiforme hypoxia microenvironment. Oncogene. 2019; 38:103–119. 10.1038/s41388-018-0423-9. 30082910 PMC6318269

[42] Cheng S , Xie W , Miao Y , Guo J , Wang J , Li C , Zhang Y . Identification of key genes in invasive clinically non-functioning pituitary adenoma by integrating analysis of DNA methylation and mRNA expression profiles. J Transl Med. 2019; 17:407. 10.1186/s12967-019-02148-3. 31796052 PMC6892283

[43] Vuori N , Sandholm N , Kumar A , Hietala K , Syreeni A , Forsblom C , Juuti-Uusitalo K , Skottman H , Imamura M , Maeda S , Summanen PA , Lehto M , Groop PH , et al. CACNB2 Is a novel susceptibility gene for diabetic retinopathy in type 1 diabetes. Diabetes. 2019; 68:2165–2174. 10.2337/db19-0130. 31439644 PMC6804633

[44] Liu F , Gong X , Yao X , Cui L , Yin Z , Li C , Tang Y , Wang F . Variation in the CACNB2 gene is associated with functional connectivity of the Hippocampus in bipolar disorder. BMC Psychiatry. 2019; 19:62. 10.1186/s12888-019-2040-8. 30744588 PMC6371424

[45] Niu Y , Gong Y , Langaee TY , Davis HM , Elewa H , Beitelshees AL , Moss JI , Cooper-DeHoff RM , Pepine CJ , Johnson JA . Genetic variation in the beta 2 subunit of the voltage-gated calcium channel (CACNB2) and pharmacogenetic association with adverse cardiovascular outcomes in the INternational VErapamil SR-Trandolapril STudy-GENEtic Substudy (INVEST-GENES). Circ Cardiovasc Genet. 2010; 3:548–555. 10.1161/CIRCGENETICS.110.957654. 21156931 PMC3060561

[46] Breitenkamp AFS , Matthes J , Nass RD , Sinzig J , Lehmkuhl G , Nürnberg P , Herzig S . Rare mutations of CACNB2 found in autism spectrum disease-affected families alter calcium channel function. PLoS One. 2014; 9:e95579. 10.1371/journal.pone.0095579. 24752249 PMC3994086

[47] Yang H , Zhang Q , He J , Lu W . Regulation of calcium signaling in lung cancer. J Thorac Dis. 2010; 2:52–56. 22263018 PMC3256429

[48] Stewart TA , Yapa KTDS , Monteith GR . Altered calcium signaling in cancer cells. Biochim Biophys Acta. 2015; 1848:2502–2511. 10.1016/j.bbamem.2014.08.016. 25150047

[49] Cui C , Merritt R , Fu L , Pan Z . Targeting calcium signaling in cancer therapy. Acta Pharm Sin B. 2017; 7:3–17. 10.1016/j.apsb.2016.11.001. 28119804 PMC5237760

[50] Hagan JP , O’Neill BL , Stewart CL , Kozlov SV , Croce CM . At least ten genes define the imprinted Dlk1-Dio3 cluster on Mouse chromosome 12qF1. PLoS One. 2009; 4:e4352. 10.1371/journal.pone.0004352. 19194500 PMC2632752

[51] Benetatos L , Hatzimichael E , Londin E , Vartholomatos G , Loher P , Rigoutsos I , Briasoulis E . The microRNAs within the DLK1-DIO3 genomic region: involvement in disease pathogenesis. Cell Mol Life Sci. 2013; 70:795–814. 10.1007/s00018-012-1080-8. 22825660 PMC11114045

[52] Cai M , Xu L , Shen L , Zhang J . [The expression of long non-coding RNA-LINC01410 in pancreatic cancer and its effect on proliferation and migration of pancreatic cancer cells]. [Article in Chinese]. Zhonghua Yi Xue Za Zhi. 2019; 99:1406–1411. 10.3760/cma.j.issn.0376-2491.2019.18.010. 31137129

[53] Zhang JX , Chen ZH , Chen DL , Tian XP , Wang CY , Zhou ZW , Gao Y , Xu Y , Chen C , Zheng ZS , Weng HW , Ye S , Kuang M , et al. LINC01410-miR-532-NCF2-NF-kB feedback loop promotes gastric cancer angiogenesis and metastasis. Oncogene. 2018; 37:2660–2675. 10.1038/s41388-018-0162-y. 29483646 PMC5955863

[54] Jiang T , Wang C , Zhu Y , Han H . LINC01410 promotes cell proliferation and migration of cholangiocarcinoma through modulating miR-124-3p/SMAD5 axis. J Gene Med. 2020; 22:e3162. 10.1002/jgm.3162. 31951299

[55] Kuja-Panula J , Kiiltomäki M , Yamashiro T , Rouhiainen A , Rauvala H . AMIGO, a transmembrane protein implicated in axon tract development, defines a novel protein family with leucine-rich repeats. J Cell Biol. 2003; 160:963–973. 10.1083/jcb.200209074. 12629050 PMC2173769

[56] Xia H , Chen J , Shi M , Gao H , Sekar K , Seshachalam VP , Ooi LLPJ , Hui KM . EDIL3 is a novel regulator of epithelial-mesenchymal transition controlling early recurrence of hepatocellular carcinoma. J Hepatol. 2015; 63:863–873. 10.1016/j.jhep.2015.05.005. 25980764

[57] Wang JP , Hielscher A . Fibronectin: how its aberrant expression in tumors may improve therapeutic targeting. J Cancer. 2017; 8:674–682. 10.7150/jca.16901. 28367247 PMC5370511

[58] Ouderkirk JL , Krendel M . Non-muscle myosins in tumor progression, cancer cell invasion and metastasis. Cytoskeleton (Hoboken). 2014; 71:447–463. 10.1002/cm.21187. 25087729 PMC4167922

[59] Weber L , Maßberg D , Becker C , Altmüller J , Ubrig B , Bonatz G , Wölk G , Philippou S , Tannapfel A , Hatt H , Gisselmann G . Olfactory receptors as biomarkers in human breast carcinoma tissues. Front Oncol. 2018; 8:33. 10.3389/fonc.2018.00033. 29497600 PMC5818398

[60] Ruan H , Li Y , Wang X , Sun B , Fang W , Jiang S , Liang C . CRYAB inhibits migration and invasion of bladder cancer cells through the PI3K/AKT and ERK pathways. Jpn J Clin Oncol. 2020; 50:254–260. 10.1093/jjco/hyz172. 31829429

[61] Reitz C , Conrad C , Roszkowski K , Rogers RS , Mayeux R . Effect of genetic variation in LRRTM3 on risk of Alzheimer disease. Arch Neurol. 2012; 69:894–900. 10.1001/archneurol.2011.2463. 22393166 PMC3391336

[62] Wang J , Yu JT , Jiang T , Tan MS , Wang HF , Tan L , Hu N , Sun L , Zhang W , Tan L . Association of LRRTM3 polymorphisms with late-onset alzheimer’s disease in Han Chinese. Exp Gerontol. 2014; 52:18–22. 10.1016/j.exger.2014.01.013. 24463050

[63] Wimmer K , Zhu XX , Lamb BJ , Kuick R , Ambros PF , Kovar H , Thoraval D , Motyka S , Alberts JR , Hanash SM . Co-amplification of a novel gene, NAG, with the N-myc gene in neuroblastoma. Oncogene. 1999; 18:233–238. 10.1038/sj.onc.1202287. 9926938

[64] Shi X , Lu L , Jin X , Liu B , Sun X , Lu L , Jiang Y . GRIN3A and MAPT stimulate nerve overgrowth in macrodactyly. Mol Med Rep. 2016; 14:5637–5643. 10.3892/mmr.2016.5923. 27840953

[65] Dhillon AS , Hagan S , Rath O , Kolch W . MAP kinase signalling pathways in cancer. Oncogene. 2007; 26:3279–3290. 10.1038/sj.onc.1210421. 17496922

[66] Jin DH , Lee J , Kim KM , Kim S , Kim DH , Park J . Overexpression of MAPK15 in gastric cancer is associated with copy number gain and contributes to the stability of c-Jun. Oncotarget. 2015; 6:20190–20203. 10.18632/oncotarget.4171. 26035356 PMC4652997

[67] Yokoyama A . RNA polymerase II-dependent transcription initiated by selectivity factor 1: a central mechanism used by MLL fusion proteins in leukemic transformation. Front Genet. 2019; 9:722. 10.3389/fgene.2018.00722. 30693017 PMC6339877

[68] Best KE , Rankin J . Long-term survival of individuals born with congenital heart disease: a systematic review and meta-analysis. J Am Heart Assoc. 2016; 5:e002846. 10.1161/JAHA.115.002846. 27312802 PMC4937249

[69] Dubé BP , Dres M . Diaphragm dysfunction: diagnostic approaches and management strategies. J Clin Med. 2016; 5:113. 10.3390/jcm5120113. 27929389 PMC5184786

[70] Friedman DN , Henderson TO . Late effects and survivorship issues in patients with neuroblastoma. Children (Basel). 2018; 5:107. 10.3390/children5080107. 30082653 PMC6111874

[71] Liu Z , Grant CN , Sun L , Miller BA , Spiegelman VS , Wang HG . Expression patterns of immune genes reveal heterogeneous subtypes of high-risk neuroblastoma. Cancers (Basel). 2020; 12:1739. 10.3390/cancers12071739. 32629858 PMC7408437

[72] Russo R , Cimmino F , Pezone L , Manna F , Avitabile M , Langella C , Koster J , Casale F , Raia M , Viola G , Fischer M , Iolascon A , Capasso M . Kinome expression profiling of human neuroblastoma tumors identifies potential drug targets for ultra high-risk patients. Carcinogenesis. 2017; 38:1011–1020. 10.1093/carcin/bgx077. 28968651

[73] Formicola D , Petrosino G , Lasorsa VA , Pignataro P , Cimmino F , Vetrella S , Longo L , Tonini GP , Oberthuer A , Iolascon A , Fischer M , Capasso M . An 18 gene expression-based score classifier predicts the clinical outcome in stage 4 neuroblastoma. J Transl Med. 2016; 14:142. 10.1186/s12967-016-0896-7. 27188717 PMC4870777

[74] Goldman M , Craft B , Hastie M , Repečka K , Kamath A , McDade F , Rogers D , Brooks AN , Zhu J , Haussler D . The UCSC Xena platform for public and private cancer genomics data visualization and interpretation. bioRxiv. 2019; 326470 10.1101/326470.

[75] Vivian J , Rao AA , Nothaft FA , Ketchum C , Armstrong J , Novak A , Pfeil J , Narkizian J , Deran AD , Musselman-Brown A , Schmidt H , Amstutz P , Craft B , et al. Toil enables reproducible, open source, big biomedical data analyses. Nat Biotechnol. 2017; 35:314–316. 10.1038/nbt.3772. 28398314 PMC5546205

[76] Robinson MD , McCarthy DJ , Smyth GK . edgeR: a Bioconductor package for differential expression analysis of digital gene expression data. Bioinformatics. 2010; 26:139–140. 10.1093/bioinformatics/btp616. 19910308 PMC2796818

[77] Love MI , Huber W , Anders S . Moderated estimation of fold change and dispersion for RNA-seq data with DESeq2. Genome Biol. 2014; 15:550. 10.1186/s13059-014-0550-8. 25516281 PMC4302049

[78] Yu G , Wang LG , Han Y , He QY . clusterProfiler: an R package for comparing biological themes among gene clusters. OMICS. 2012; 16:284–287. 10.1089/omi.2011.0118. 22455463 PMC3339379

[79] Yu G , Wang LG , Yan GR , He QY . DOSE: an R/Bioconductor package for disease ontology semantic and enrichment analysis. Bioinformatics. 2015; 31:608–609. 10.1093/bioinformatics/btu684. 25677125

[80] Pedregosa F , Varoquaux G , Gramfort A , Michel V , Thirion B , Grisel O , Blondel M , Prettenhofer P , Weiss R , Dubourg V , Vanderplas J , Passos A , Cournapeau D . Scikit-learn: Machine Learning in Python. J Mach Learn Res. 2011; 12:2825–2830.

[81] Chang CC , Lin CJ . LIBSVM: A library for support vector machines. ACM Trans Intell Syst Technol. 2011; 2:27 10.1145/1961189.1961199.

[82] Huynh-Thu VA , Irrthum A , Wehenkel L , Geurts P . Inferring regulatory networks from expression data using tree-based methods. PLoS One. 2010; 5:e12776. 10.1371/journal.pone.0012776. 20927193 PMC2946910

[83] Shannon P , Markiel A , Ozier O , Baliga NS , Wang JT , Ramage D , Amin N , Schwikowski B , Ideker T . Cytoscape: a software environment for integrated models of biomolecular interaction networks. Genome Res. 2003; 13:2498–2504. 10.1101/gr.1239303. 14597658 PMC403769

