# Supplementary material for: Identification of novel prognostic markers of survival time in high-risk neuroblastoma using gene expression profiles
Source: Oncotarget. 2020 Nov 17;11(46):4293–305. doi: 10.18632/oncotarget.27808 (PMC7679032; doi:10.18632/oncotarget.27808)
Supplement: Supplementary file 1 [file oncotarget-11-4293-s001.pdf]

Identification of novel prognostic markers of survival time in high-risk neuroblastoma using gene expression profiles

SUPPLEMENTARY MATERIALS

Supplementary Text 1: The 1018 and 680 GENIE3 weighted interactions of DEGs in the SS and LS groups, respectively. See Supplementary Text 1
